# Spiking neural networks fine-tuning for brain image segmentation

**DOI:** 10.3389/fnins.2023.1267639

**Published:** 2023-11-01

**Authors:** Ye Yue, Marc Baltes, Nidal Abuhajar, Tao Sun, Avinash Karanth, Charles D. Smith, Trevor Bihl, Jundong Liu

**Affiliations:** ^1^School of Electrical Engineering and Computer Science, Ohio University, Athens, OH, United States; ^2^Centrum Wiskunde and Informatica (CWI), Machine Learning Group, Amsterdam, Netherlands; ^3^Department of Neurology, University of Kentucky, Lexington, KY, United States; ^4^Department of Biomedical, Industrial and Human Factors Engineering, Wright State University, Dayton, OH, United States

**Keywords:** spiking neural network (SNN), ANN-SNN conversion, image segmentation, fine-tuning, U-Net

## Abstract

**Introduction:**

The field of machine learning has undergone a significant transformation with the progress of deep artificial neural networks (ANNs) and the growing accessibility of annotated data. ANNs usually require substantial power and memory usage to achieve optimal performance. Spiking neural networks (SNNs) have recently emerged as a low-power alternative to ANNs due to their sparsity nature. Despite their energy efficiency, SNNs are generally more difficult to be trained than ANNs.

**Methods:**

In this study, we propose a novel three-stage SNN training scheme designed specifically for segmenting human hippocampi from magnetic resonance images. Our training pipeline starts with optimizing an ANN to its maximum capacity, then employs a quick ANN-SNN conversion to initialize the corresponding spiking network. This is followed by spike-based backpropagation to fine-tune the converted SNN. In order to understand the reason behind performance decline in the converted SNNs, we conduct a set of experiments to investigate the output scaling issue. Furthermore, we explore the impact of binary and ternary representations in SNN networks and conduct an empirical evaluation of their performance through image classification and segmentation tasks.

**Results and discussion:**

By employing our hybrid training scheme, we observe significant advantages over both ANN-SNN conversion and direct SNN training solutions in terms of segmentation accuracy and training efficiency. Experimental results demonstrate the effectiveness of our model in achieving our design goals.

## 1. Introduction

The advancement of Artificial Neural Networks (ANNs) has revolutionized many AI-related domains, delivering state-of-the-art results across a wide range of tasks in computer vision and medical image analysis. However, the exceptional performance of ANNs often comes at the cost of significant computational requirements, limiting their practicality in power-hungry systems like edge devices and portable gadgets. In recent years, spiking neural networks (SNNs) have emerged as a promising low-power alternative to ANNs. SNNs replicate the temporal and sparse spiking behavior exhibited by biological neurons (Roy et al., 2019; Davies et al., 2021; Manna et al., 2022, 2023; Vicente-Sola et al., 2022). Unlike traditional neural networks, SNN neurons consume energy only during spike generation, leading to sparser activations and natural enhancements in Size, Weight, and Power (SWaP) characteristics.

An SNN can be trained directly from scratch, utilizing a certain surrogate gradient function for backpropagation. Due to the binary nature of the signals, training SNNs is often more challenging and time-consuming compared to training ANNs, especially for networks with complex structures (Shrestha and Orchard, 2018; Wu et al., 2018; Rathi et al., 2020; Li et al., 2021). Moreover, for many pre-trained ANNs on large datasets, e.g., ImageNet or LibriSpeech, training equivalent SNNs from scratch would be very difficult.

Alternatively, an SNN can be obtained by converting from a fully trained ANN. Many state-of-the-art ANN-SNN conversion solutions (Diehl et al., 2015; Rueckauer et al., 2016; Sengupta et al., 2019; Ho and Chang, 2021) focus on setting proper firing thresholds after copying the weights from a trained ANN model. The converted SNNs commonly require a large number of time steps to achieve comparable performance.

Furthermore, most existing SNN works focus on recognition related tasks. Image segmentation, a very important task in medical image analysis, is rarely studied, except for Patel et al. (2021) and Kim et al. (2022).

In Kim et al. (2022), take a direct training approach, which inevitably suffers from the common drawbacks of this category. Patel et al. (2021) use leaky *integrate-and-fire* (LIF) neurons for both ANNs and SNNs. While convenient for conversion, the ANN networks are limited to a specific type of activation functions and must be trained from scratch.

In this paper, we propose a three-stage SNN training scheme and apply it to segment the human hippocampus from magnetic resonance (MR) images. In our proposed pipeline, a segmentation network is first trained, followed by an ANN-SNN conversion step to initialize the weights and layer thresholds in the SNN. Then, we apply a spike-based fine-tuning process to adjust the network weights in dealing with potentially suboptimal thresholds set by the conversion.

To evaluate the effectiveness of our proposal pipeline, we conduct extensive comparisons with conversion-only and direct training methods, and demonstrate our approach can significantly improve segmentation accuracy, as well as decreases the training effort for convergence. We also carry out experiments to explore the mechanisms behind the performance drop in ANN-SNN conversion. In addition, we conduct a comparative analysis between binary (2-value) and ternary (3-value) representations in SNNs. While SNN hardware naturally uses binary spikes, ternary spikes can be resulted from the utilization of zero-mean normalization in data preprocessing and *rate coding* in spiking networks.

We choose the hippocampus as the target brain structure as accurate segmentation of hippocampus provides a quantitative foundation for many other analyses, and therefore has long been an important task in neuro-image research. A modified U-Net (Ronneberger et al., 2015) is used as the baseline ANN model in our work. This submission builds upon and expands our research previously presented at ISBI'23 (Yue et al., 2023).

The contributions made in this study can be summarized as follows:

1. We propose a novel three-stage SNN training scheme designed specifically for segmenting human hippocampi from magnetic resonance images. To the best of our knowledge, our model is the first three-stage SNN fine-tuning work proposed for the image segmentation task, as well as on U-shaped networks.
2. Our proposed pipeline achieves comparable or better results than both full conversion methods and direct training methods, with significantly fewer time steps and much faster convergence.
3. We delve to understand the accuracy drop of ANN-SNN conversion through a set of experiments to investigate the output scaling issue. Furthermore, we explore the impact of binary and ternary representations in SNN networks. We believe our observations and conclusions are valuable in furthering the understanding of SNN training.

## 2. Background and related work

### 2.1. Hippocampus segmentation

In various neuroimage studies, segmenting brain structures from magnetic resonance (MR) images is of great importance as it often impacts the outcomes of subsequent analysis steps. Among the anatomical structures, the hippocampus holds particular interest due to its crucial role in memory formation. Moreover, it is one of the brain regions susceptible to tissue damage in Alzheimer's Disease. Traditional approaches to automatic hippocampal segmentation include atlas-based and patch-based methods (Coupé et al., 2011; Tong et al., 2013; Song et al., 2015), which commonly rely on identifying similarities between the target image and anatomical atlases to infer labels for individual voxels.

In recent years, deep learning models, particularly the U-net model introduced by Ronneberger et al. (2015) and its variations, have emerged as dominant solutions for medical image segmentation. In our research, we have developed two network-based approaches for hippocampus segmentation, as outlined in Chen et al. (2017a) and Chen et al. (2017b), which have achieved state-of-the-art results. In Chen et al. (2017a), we proposed a multi-view ensemble convolutional neural network (CNN) framework in which multiple decision maps generated along different 2D views are integrated. In Chen et al. (2017b), an end-to-end deep learning architecture is developed that combines CNN and recurrent neural network (RNN) to better leverage the dimensional anisotropism in volumetric medical data.

### 2.2. Training spiking neural networks

The training of ANN models predominantly relies on gradient descent and backpropagation. Unfortunately, the neurons in SNNs are highly non-differentiable with a large temporal aspect. This makes gradient descent much more difficult to apply. As a result, workaround approaches have been proposed, which can be roughly grouped two categories: direct training solutions and ANN-SNN conversion solutions.

The direct training approach employs surrogate gradients (Wu et al., 2018; Kim and Panda, 2020), which serve as approximations of the step function, enabling the backpropagation algorithm to update the network weights. In order to assign spatial and temporal gradients to neurons, spike-timing-dependent plasticity (STDP) is commonly utilized. STDP actively adjusts connection weights based on the firing timing of associated neurons, providing a mechanism for learning in SNNs, as explored by Liu et al. (2020).

Training an ANN first and subsequently converting it into an SNN offers a viable solution to bypass the non-differentiability issue. One major group of conversion solutions (Diehl et al., 2015; Rueckauer et al., 2016; Sengupta et al., 2019; Ho and Chang, 2021) involve training ANNs with rectified linear unit (ReLU) neurons and then converting them into SNNs with integrate-and-fire (IF) neurons by appropriately setting firing thresholds. An alternative method proposed by Hunsberger and Eliasmith (Hunsberger and Eliasmith, 2015; Rasmussen, 2019) utilizes soft LIF neurons, which incorporate smoothing operations around the firing threshold. This smoothing enables gradient-based backpropagation to be applied during network training. Consequently, the conversion process from ANN to SNN becomes relatively straightforward.

### 2.3. SNN models for semantic image segmentation

Patel et al. (2021) and Kim et al. (2022) are two major SNN models proposed for semantic image segmentation. In Kim et al. (2022), the authors investigate ANN-SNN conversion and surrogate gradient learning. The results from this study show that direct training with surrogate gradient learning achieves lower latency and higher performance compared to ANN-SNN conversion.

In Patel et al. (2021), an SNN U-Net is converted from an ANN using the Nengo framework. Both rate-based ANN and spike-based SNN are trained and evaluated using a modified version of the ISBI 2D EM Segmentation cell dataset. While they demonstrate slightly worse performance compared to the TensorFlow baseline model, both the Nengo ANN and the converted SNN achieve similar segmentation accuracy using the same number of neurons and weights. This similarity should be partly attributed to the fact that Nengo ANNs and converted SNNs utilize the same type of LIF neurons.

## 3. Method

In this study, we propose a three-stage training framework for optimizing U-shaped SNNs. Our approach diverges from the two prevalent strategies: ANN-SNN conversion and direct training. The major advantage of ANN-SNN conversion lies in the fact that ANNs are easy to be trained to their maximum capacities. The downside of conversion is that the procedure requires a large number of time-steps to obtain fully converted SNNs. Direct trainings of SNNs have the benefit of being straightforward, but they are generally more difficult and time-consuming than trainings of ANNs, primarily due to the binary nature of spiking signals.

Our three-stage approach is designed to harness the strengths of both strategies while avoiding their limitations. In our pipeline, an ANN is first fully trained, before being converted to an SNN. We use *quick* (or early-stop) conversions, bypassing the lengthy full conversion procedures. While the performance of these quick-converted SNNs is not comparable to the original ANNs, it nevertheless produces well-initialized SNNs to be fine-tuned. With good initializations, the fine-tuning step can bring SNNs' performance back to near-ANN levels. This is the primary motivation and novelty of our design. To the best of our knowledge, it is the first such design on U-shaped spiking networks, as well as for image segmentation.

**Figure 1:**
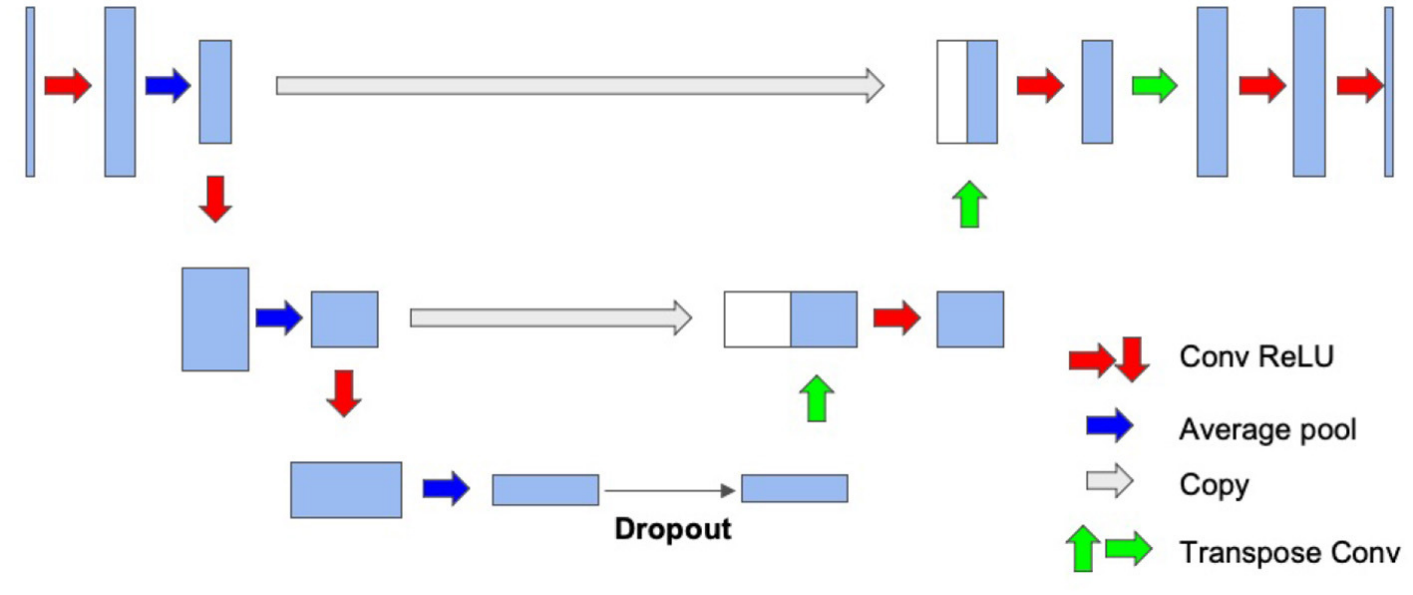
Network architecture of our baseline ANN model.

We adopt a modified U-Net architecture as the baseline ANN model. The architecture follows an encoding and decoding structure, as illustrated in Figure 1. Taking 2D images as inputs, the encoding component consists of a series of convolutional layers followed by pooling layers to extract high-level latent features. On the other hand, the decoder utilizes transpose or deconvolution layers to reconstruct the segmentation ground-truth mask. To leverage local information effectively, *skip connections* are introduced to concatenate the corresponding feature maps between the encoding and decoding stacks.

In order to accommodate the constraints associated with ANN-SNN conversion, several modifications are made to the original U-Net and our previous hippocampus segmentation network (Chen et al., 2017a). Firstly, we substitute max-pooling with average-pooling since effective implementation of max-pooling in SNNs is challenging. Secondly, we eliminate the bias components of the neurons, as they may interfere with the voltage thresholds in SNNs, making the training process more complex. Thirdly, batch normalizations are removed due to the absence of bias terms. Lastly, *dropout layers* are added to provide regularization for both ANN and SNN trainings.

### 3.1. Our proposed three-stage SNN fine-tuning model

Rathi et al. (2020) proposed an SNN fine-tuning solution for the image classification task. In their study, three models are compared: (1) *full* ANN-SNN conversion methods; (2) direct training SNNs from scratch; and (3) fast ANN-SNN conversions, followed by fine-tuning the converted SNNs. For full ANN-SNN conversions, they take time-step of 2,500 for all models, while *fast* conversions use fewer than 250 time-steps. Their study demonstrates that the “fast conversion + fine-tuning” approach can achieve, with much fewer time steps, similar accuracy compared to fully converted SNNs, as well as faster convergence than direct training methods.

Inspired by Rathi et al. (2020), we develop a three-stage SNN fine-tuning scheme for semantic segmentation. We first train a segmentation ANN to its full convergence and then convert it to a spiking network with greatly reduced time steps, which we call *early-stop* conversion. The converted SNN is then taken as an initial state for a fine-tuning procedure. In summary, our three-stage segmentation pipeline consists of ANN training → early-stop conversion → SNN fine-tuning steps.

**The SNN neuron** model in our work is integrate-and-fire (IF) model where the membrane potential will not decrease during the time when neuron does not fire as opposed to LIF neurons.

The dynamics of our IF neurons can be described as:


(1) $$\begin{array}{l} u_{i}^{t}=u_{i}^{t-1}+\underset{j}{\sum }w_{ij}o_{j}-vo_{i}^{t-1} \end{array}$$

(2) $$o_{i}^{t-1}=\{\begin{array}{ll} 1 & \text{if }u_{i}^{t-1}>v\\ 0 & \text{otherwise} \end{array}$$

where *u* is the membrane potential, *t* is the time step, subscript *i* and *j* represent the post- and pre-neuron, respectively, *w* is the weight connecting the pre- and post-neuron, *o* is the binary output spike, and *v* is the firing threshold. Each neuron integrates the inputs from the previous layer into its membrane potential, and reduces the potential by a threshold voltage if a spike is fired.

Our SNN network has the same architecture as the baseline ANN, where the signals transmitted within the SNN are rate-coded spike trains generated through a Poisson generator. During the conversion process, we load the weights of the trained ANN into the SNN network and set the thresholds for all layers to 1 s. Then, a threshold balancing procedure (Sengupta et al., 2019) is carried out to update the threshold of each layer.

**Fine-tuning** of the converted SNN is conducted using spike-based backpropagation. It starts at the output layer, where the signals are continuous membrane potentials, generated through the summation:


(3) $$\begin{array}{l} u_{i}^{t}=u_{i}^{t-1}+\underset{j}{\sum }w_{ij}o_{j} \end{array}$$

The number of neurons in the output layer is the same as the size of the input image. Compared with the hidden layer neurons in Equation (1), the output layer does not fire and therefore the voltage reduction term is removed. Each neuron in the output layer is connected to a Sigmoid activation function to produce the predictive probability of the corresponding pixel belonging to the target area (hippocampus).

Let *L*(·) be the loss function defined based on the ground-truth mask and the predictions. In the output layer, neurons do not generate spikes and thus do not have the non-differentiable problem. The update of the hidden layer parameters *W*_*ij*_ is described by:


(4) $$\begin{array}{l} \Delta W_{ij}=\underset{t}{\sum }\frac{\partial L}{\partial W_{ij}^{t}}=\underset{t}{\sum }\frac{\partial L}{\partial o_{i}^{t}}\frac{\partial o_{i}^{t}}{\partial u_{i}^{t}}\frac{\partial u_{i}^{t}}{\partial W_{ij}^{t}} \end{array}$$

Due to the non-differentiability of spikes, a surrogate gradient-based method is used in backpropagation. In Rathi et al. (2020), the authors propose a surrogate gradient function $\frac{\partial o^{t}}{\partial u^{t}}=\alpha e^{-\beta \Delta t}$. In this work, we choose a linear approximation proposed in Bellec et al. (2018), which is described as:


(5) $$\begin{array}{l} \frac{\partial o^{t}}{\partial u^{t}}=\alpha \max\left\{0,1-|u^{t}-V_{t}|\right\} \end{array}$$

where *V*_*t*_ is the threshold potential at time *t*, and α is a constant.

As demonstrated in Rathi model, we hope our three-stage pipeline can achieve comparable or better accuracy, but with much fewer time steps compared to full conversion methods, as well as much faster convergence than direct training methods. It should be noted that our work is the first attempt of exploring the application of spike-based fine-tuning and threshold balancing on fully convolutional networks (FCNs), including the U-Net.

### 3.2. Different losses

We explore different loss functions in this work, which include *binary cross entropy* (BCE), *Dice loss* and a combination of the two losses (BCE-Dice). BCE loss is the average of per-pixel loss and gives an unbiased measurement of pixel-wise similarity between prediction and ground-truth:


(6) $$\begin{array}{l} L_{\text{BCE}}=\overset{N}{\underset{i=1}{\sum }}-\left[r_{i}\log s_{i}+\left(1-r_{i}\right)\log\left(1-s_{i}\right)\right] \end{array}$$

where *s*_*i*_∈[0, 1] is the predicted value of a pixel and *r*_*i*_∈{0, 1} is the ground-truth label for the same pixel. ϵ is a small number to smooth the loss, which is set to 10^−5^ in our experiments. *Dice loss* focuses more on the extent to which the predicted and ground-truth overlap:


(7) $$L_{\text{Dice}}=\frac{2\sum_{i}s_{i}r_{i}+\epsilon }{\sum_{i}s_{i}+\sum_{i}r_{i}+\epsilon }$$

We also explore the effects of a weighted combination of BCE and Dice: *L*_BCE_Dice_ = 0.3 × *L*_BCE_+0.7 × *L*_Dice_.

### 3.3. Binary (2-value) SNNs vs. ternary (3-value) SNNs

Data normalization is a common pre-processing step in machine learning, where samples are scaled into a specific range, such as [0, 1] or [−1, 1]. The latter approach, known as zero-mean normalization, involves centering the data around zero, which is often helpful to eliminate biases and improve convergence in optimization procedures.

In spiking networks, rate coding is a widely used encoding scheme to convert input values into firing rates of spiking neurons. This process often employs *Poisson* generators, which sample from a uniform distribution at each time step and compare the sampled number with a target value. Taking a grayscale image as an example: if the maximum intensity is 255 and a pixel has an intensity of 10, then the target value is 10/255. If the sampled number is less than the target value, a spike is emitted; otherwise, no spike is emitted (Diehl et al., 2015; Hazan et al., 2018).

**Figure 2:**
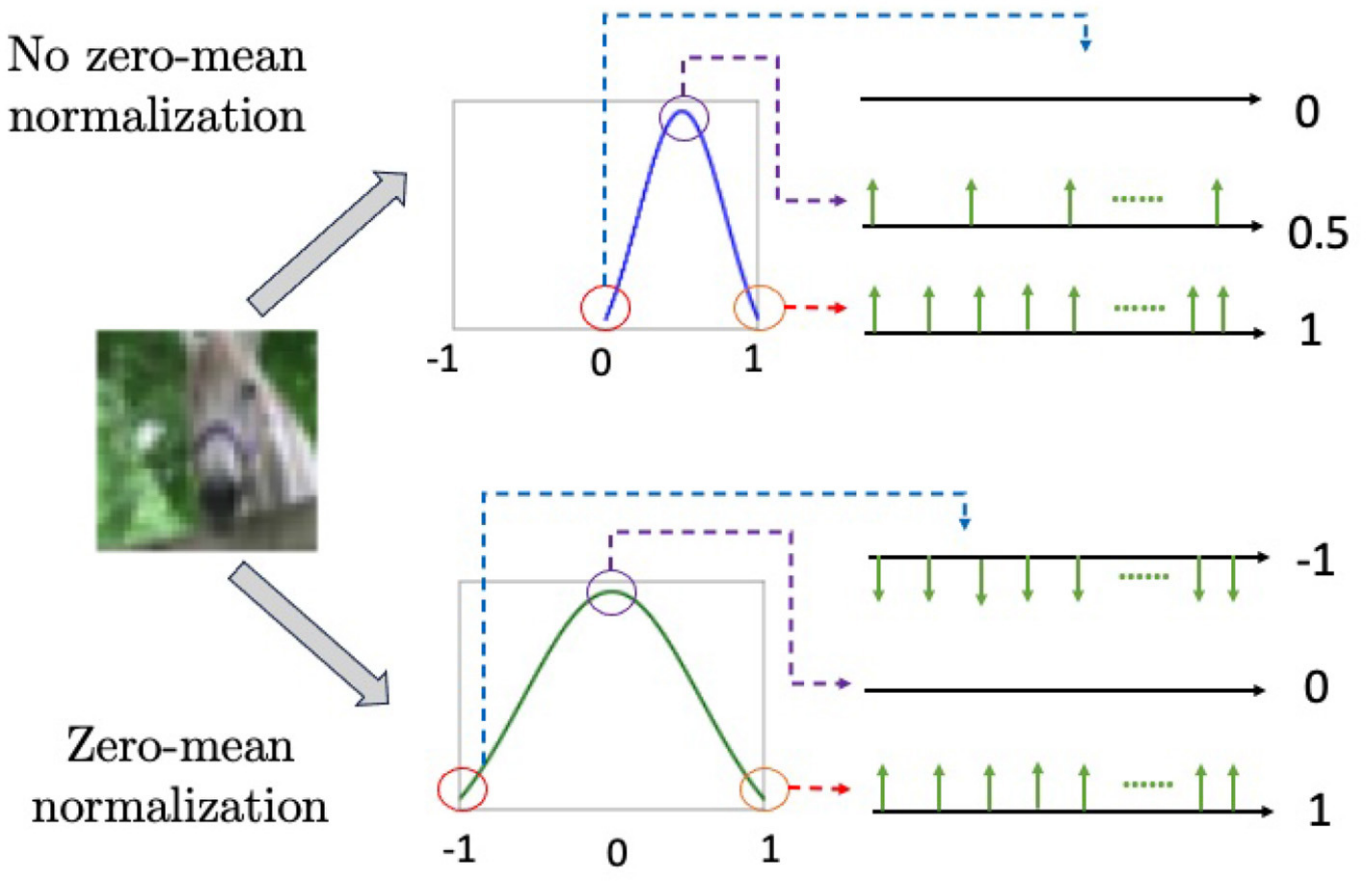
The combination of zero-mean normalization and Poisson generators produces negative spikes, as shown in the bottom figure. The upper figure illustrates [0, 1] normalization, which does not generate negative spikes.

Zero-mean normalization can result in negative values. To represent negative values in SNNs, one approach is to introduce negative spikes, where firing is determined by the comparisons of absolute values and the sign of the target value is assigned to the fired spikes. As a result, the range of values for a single spike expands from {0, 1} (i.e., binary spikes) to {−1, 0, 1} (ternary spikes), as in Perez-Carrasco et al. (2013) and Rathi et al. (2020). Figure 2 illustrates the difference between ternary spikes, sampled from a zero-mean normalization and binary spikes from a [0, 1] normalization.

While zero-mean normalization and negative spikes offer convenience in training SNNs at the software level, directly implementing negative spikes into the hardware is often quite challenging. In this work, we investigate the performance discrepancies between binary and ternary SNNs based on image classification and segmentation tasks. Detailed results will be presented in the next section.

## 4. Experiments and results

In this work, we carry out two sets of experiments. The first set is for hippocampus segmentation, and comparisons are conducted between our proposed SNN fine-tuning scheme with two competing solutions: *full conversion* method and *direct training* method. The second set is to evaluate the performance disparity between 2-value and 3-value SNNs, using image classification and semantic segmentation settings.

### 4.1. Data and training

The segmentation data were obtained from the ADNI database (https://adni.loni.usc.edu/) and extracted in our previous work (Chen et al., 2017a,b). We downloaded a total of 110 baseline T1-weighted whole brain MRI images from different subjects, along with their corresponding hippocampus masks. In this study, we only included normal control subjects. Given that the hippocampus accounts for a small fraction of the entire brain and is generally situated in a rather consistent position, we approximated and cropped the right hippocampus of each subject. These cropped sections served as the inputs for segmentation. The dimensions of the cropping box are 24 × 56 × 48.

We utilize a five-fold cross-validation method to train and evaluate our proposed fine-tuning model. For each fold, the test and training sets include 22 and 88 subjects, respectively. The batch size in both training and testing, for ANNs and SNNs, is set at 26. The batch size is primarily determined by the GPU and system memory where our model is trained. Our GPU is a Nvidia RTX 3080 with 10G memory and the system has 8G memory. Training of both ANN and the SNN networks uses the Adam optimizer with slightly different parameters. We set the initial learning rate for training both networks at 0.001. This rate is later fine-tuned by the ReduceLROnPlateau scheduler in PyTorch, which monitors the loss during training and decreases the learning rate when the loss ceases to fall.

Following a similar setup in Rathi et al. (2020), we use time steps of 200 for our early-stop ANN-SNN conversion routine. The validity of this choice is substantiated through comparative experiments on different timesteps, which will be presented later in this section. The ANN models were trained with 100 epochs and SNN models were trained over 35 epochs. Also, we repeat the same ANN → early-stop conversion → fine-tuning procedure with three different loss functions: BCE only, Dice only and combined BCE and Dice.

### 4.2. Segmentation results

In this section, we present and evaluate the experimental results for the proposed model. Two different performance metrics, 3D Dice ratio and 2D slice-wise Dice ratio, were used to measure the accuracy of the segmentation models. The 3D Dice ratio was calculated subject-wise for each 3D volume. Mean and standard deviation averaged from five-folds are reported. The 2D slice based Dice ratio was calculated slice by slice, and the mean and standard deviation were averaged from all test subjects' slices.

Accuracies of the model on the test data are summarized in Table 1. The best performance for the ANN and fine-tuned SNN are highlighted with bold font. It is evident that network accuracies drop significantly after conversion (middle column) and our fine-tuning procedure can bring the performance of SNNs (right column) back close to the ANN level. The models built on the three loss functions have comparable performance in ANNs and fine-tuned SNNs, with Dice loss has slight edge over BCE and BCE-Dice combined.

**Table 1:** Average accuracies (Dice scores) of ANNs, early-stop converted SNNs and fine-tuned SNNs built on three difference loss functions.

**Loss**	**ANN accuracy (Dice score)**		**Converted-SNN accuracy**		**Fine-tuned SNN accuracy**	
	**2D**	**3D**	**2D**	**3D**	**2D**	**3D**
BCE	77.76 ± 2.40	83.17 ± 1.44	30.51 ± 9.93	21.60 ± 12.39	76.92 ± 3.77	81.83 ± 2.99
Dice	**78.86 ± 2.39**	**84.21 ± 1.66**	61.78 ± 3.32	65.90 ± 6.94	**78.09 ± 3.85**	81.86 ± 4.40
BCE-Dice	78.58 ± 2.44	83.14 ± 1.59	52.03 ± 9.91	52.78 ± 14.73	77.72 ± 3.69	**81.95 ± 3.24**

**Figure 3:**
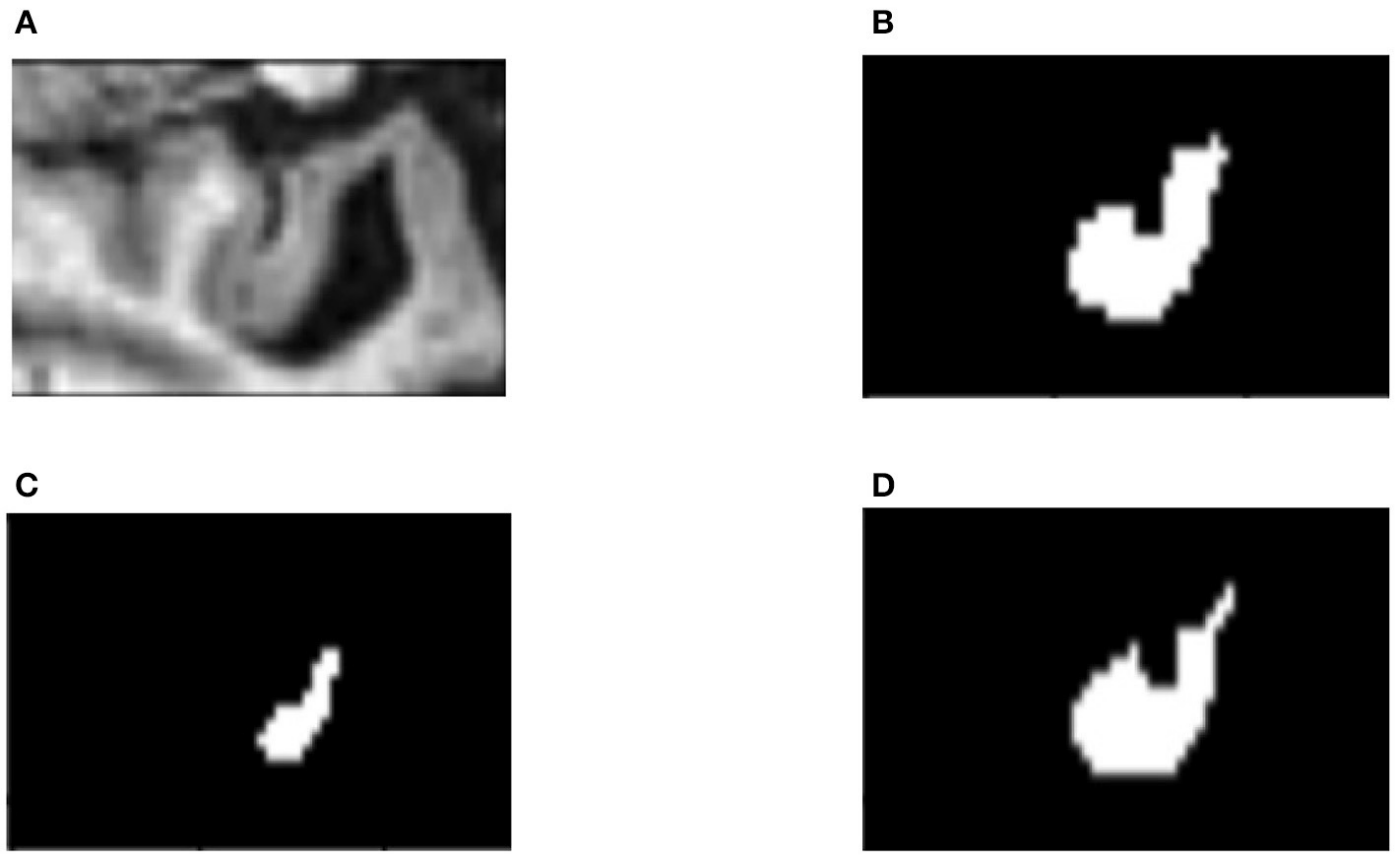
An example slice of **(A)** input; **(B)** ground-truth mask; **(C)** segmentation result from the converted SNN; and **(D)** result from the fine-tuned SNN.

**Figure 4:**
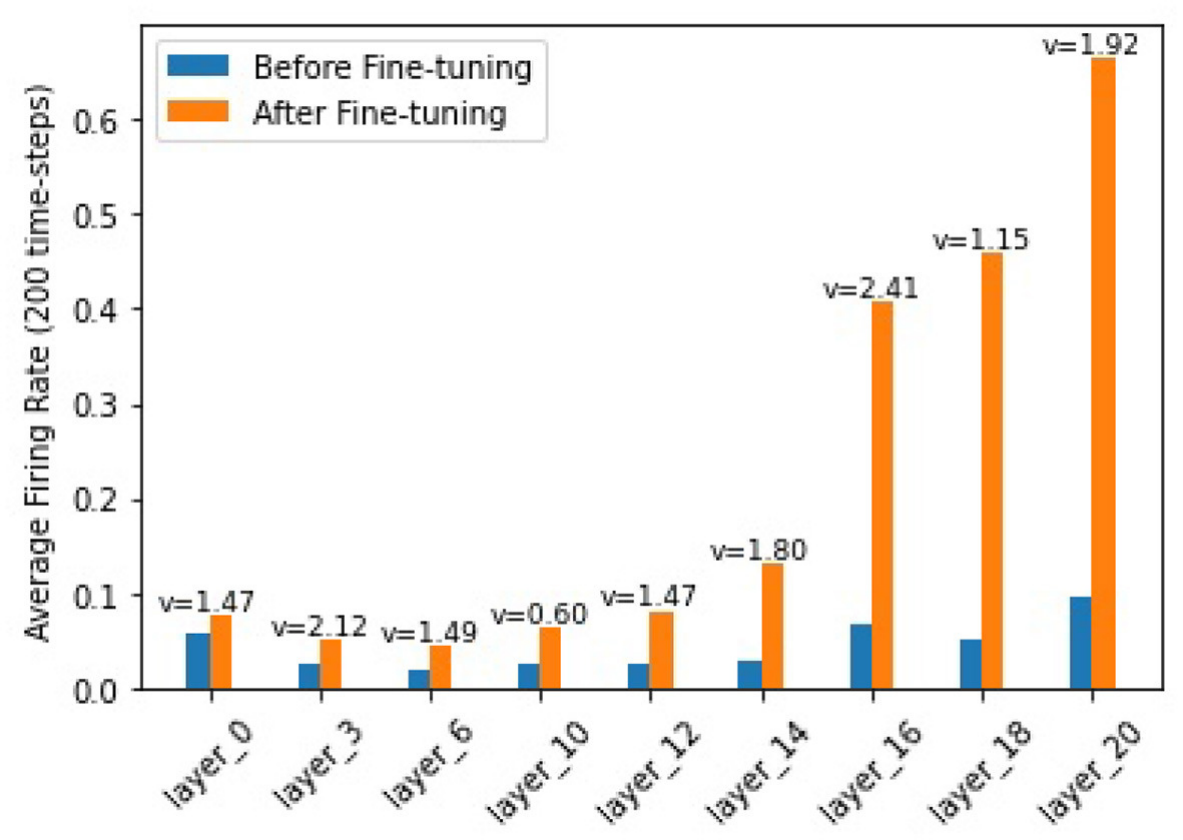
Firing frequencies of neurons in different layers. Blue bars show those for a converted-SNN and orange bars are for the fine-tuned SNN. *v* is the layer threshold.

In order to find out how the fine-tuning procedure improves the segmentation accuracy, we look into details of both the outputs and the internal spiking patterns of the networks. Figure 3 shows an example of input slice, ground-truth mask and the corresponding outputs from the converted and fine-tuned SNNs. We can see the output mask from the converted SNN (Figure 3C), while carrying a similar shape, is much smaller than the ground-truth. We believe the reason is that many neurons are not sufficiently activated due to the suboptimal thresholds set by the conversion procedure. The proposed fine-tuning step, on the other hand, can update the network weights to adjust for such thresholds, bringing the neurons back to active for improved accuracy. To confirm this thought, we record the firing frequency of each layer in the SNN models before and after the fine-tuning and plot them in Figure 4. It is evident that neurons become more active after the fine-tuning, producing more accurate segmentation predictions.

### 4.3. Investigation of the performance drop in converted SNNs

From Table 1, it is evident that converted SNNs show substantial accuracy declines compared to the original ANNs. We formulate several hypotheses for these reductions, with the simplest one being that a learned threshold at the output layer can help to properly set up the threshold for pixel labeling (Rueckauer et al., 2016; Ho and Chang, 2021).

In our implementation, the ANN's output layers yield probabilities indicating if pixels belong to the Hippocampus. These probabilities are then translated into binary values based on a threshold of 0.5 (a value above 0.5 suggests that a pixel more likely belongs to the Hippocampus than otherwise). The converted SNNs follow this same setup to determine each pixel's label. Despite efforts to ensure that every layer of the SNNs replicates the output of its corresponding ANN layer, scaling discrepancies are inevitable. These discrepancies might be a major factor for the performance drop in the converted SNNs. Such scaling may be non-linear, impacting different neurons in varied ways across layers. However, if the conversion results in a simple global shift in probabilities, adjusting the threshold could counteract the effect.

**Figure 5:**
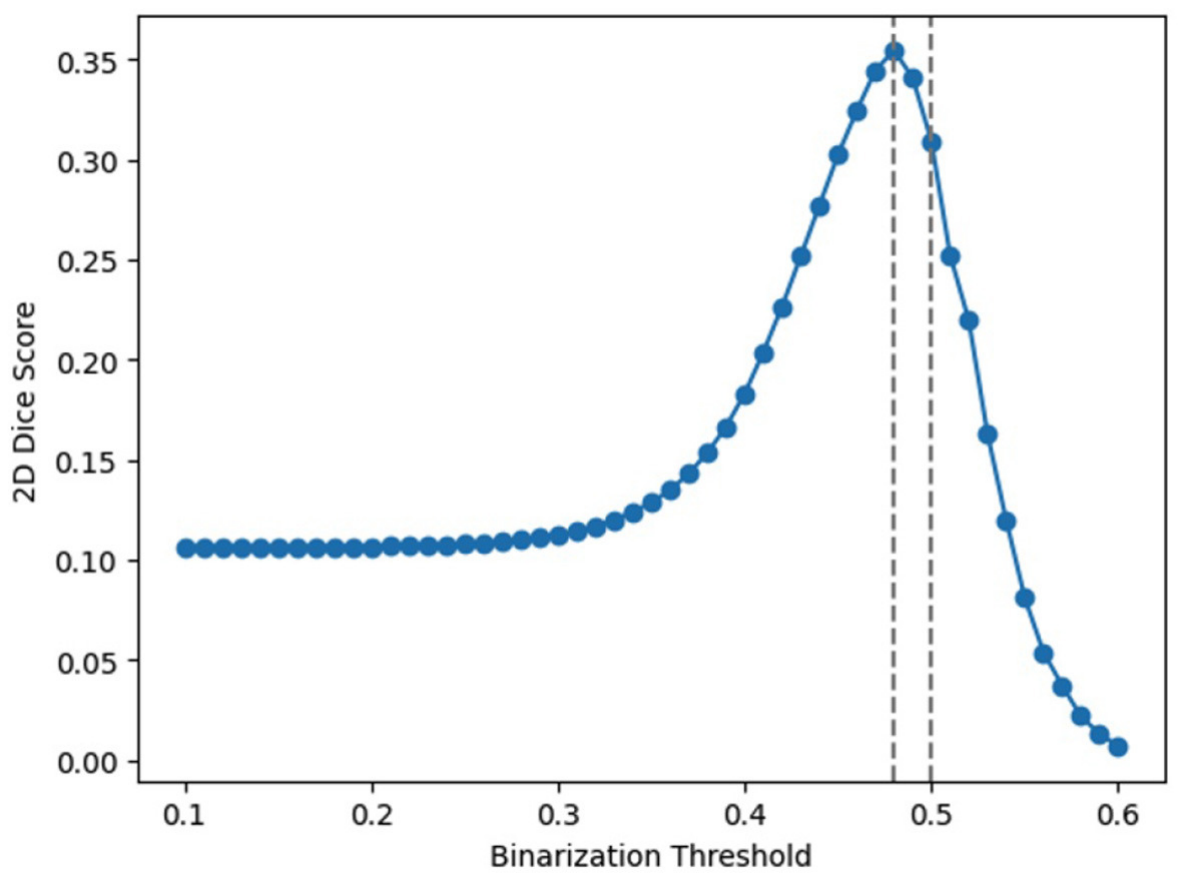
Average segmentation accuracies with different thresholds at the output layer.

To explore this, we design an experiment that employs an array of thresholds for class cut-off. Specifically, we test thresholds ranging from 0.1 to 0.6 at the output layer. The average accuracies over the entire dataset are presented in Table 2, and Figure 5 visualizes these findings. From the table, a threshold of 0.48 yielded the optimal result, which is rather close to the default 0.5 threshold. It should note that adjusting the threshold is equivalent to scaling the output; for example, using 0.2 as the threshold is equivalent to enlarging the outputs 2.5 times and then applying the 0.5 threshold. Our observations imply that a mere linear adjustment at the end does not solve the scaling issues. On the other hand, it indicates that scaling's have happened across different neurons in a non-linear manner, highlighting the need for fine-tunings SNNs after conversions.

**Table 2:** Average segmentation accuracies with different thresholds at the output layer.

**Threshold**	**0.43**	**0.44**	**0.45**	**0.46**	**0.47**	**0.48**	**0.49**	**0.50**	**0.51**	**0.52**	**0.53**	**0.54**
Accuracy (Dice score)	0.25	0.27	0.30	0.32	0.34	0.35	0.34	0.31	0.25	0.21	0.16	0.12

### 4.4. Comparisons with *full conversion* and *direct training* methods

**Figure 6:**
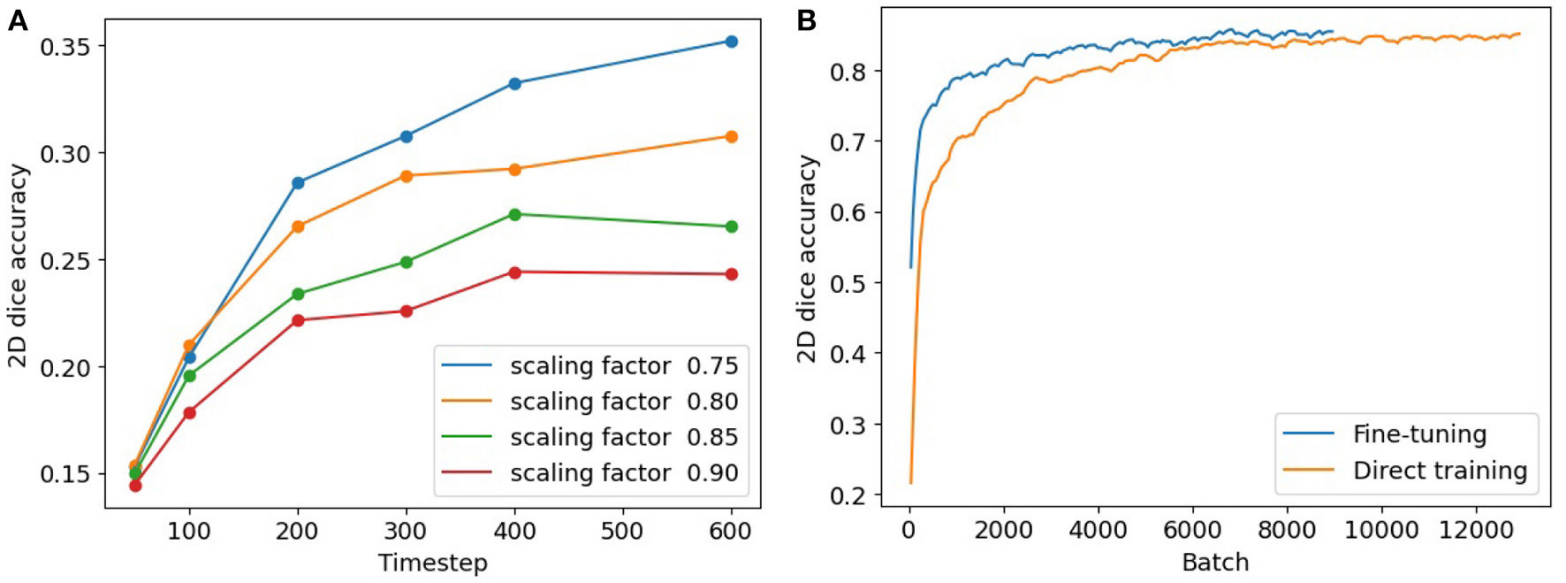
**(A)** Segmentation accuracies of converted SNN models with timesteps and scaling thresholds. **(B)** Segmentation accuracies using our fine-tuning model (in blue) and direct training (in orange).

To conduct a comparative analysis between our 3-stage fine-tuning approach and the conversion method, we follow the methodology outlined in Rathi et al. (2020). The conversion step in our 3-stage model is an early-stop process, requiring us to identify a suitable timestep to terminate the conversion in its early stage. To determine an appropriate timestep, we conduct experiments and analyze the network performance with various combinations of timestep and weight scaling. Figure 6A illustrates the segmentation accuracies achieved in these scenarios. It is noteworthy that, across all the curves, the 200th timestep consistently emerges as an *elbow point*. Beyond this point, the accuracies continue to rise, but at a significantly reduced pace. This suggests that 200 can serve as an appropriate timestep to terminate the ANN-SNN conversion and start the SNN fine-tuning procedure.

To compare our fine-tuning model with the direct training method, we track the 2D segmentation accuracies for both procedures throughout the training process. The results are summarized in Table 3. The columns in the table indicate different batch numbers and their corresponding Dice scores derived from both methods, with the results being averaged over multiple trials. Figure 6B provides a visualization of Table 3, displaying the convergence of the direct training method in orange and our fine-tuning SNN in blue. Clearly, our fine-tuning approach achieves much faster convergence and higher segmentation accuracy compared to the direct method.

**Table 3:** Segmentation accuracies (2D Dice scores) from our SNN fine-tuning and the direct training methods.

**Model**	**Number of batches**								
	**40**	**2000**	**4000**	**6000**	**8000**	**9216**	**10000**	**12000**	**13176**
Direct training	0.0147	0.7452	0.7883	0.8364	0.8286	0.8486	0.8475	0.8541	0.8530
Fine tuning	**0.2236**	**0.8114**	**0.8147**	**0.8488**	**0.8428**	**0.8635**	-	-	-

The above experiments and comparative analysis have clearly demonstrated the advantages of our three-stage approach over the traditional ANN-SNN conversion and direct training methods. In addition, for many pretrained ANNs on large datasets like ImageNet or LibriSpeech, our three-stage fine-tuning would be more pragmatic than training equivalent SNNs from scratch. Given these evident benefits, we would advocate for our three-stage pipeline to be used as a standard paradigm for SNN training.

However, our methodology is not without its constraints, especially from the applicability perspective. First, an effective ANN-SNN conversion implementation is required to utilize our strategy. Without this conversion routine in place, direct training might be the only choice. Second, the conversion and fine-tuning processes make a trade-off: a lengthier conversion reduces fine-tuning time and vice versa. Finding an optimal balance could be challenging. Lastly, the fine-tuning step in our pipeline, in nature, is a direct training with good initializations. Therefore, it also suffers from the inherent drawbacks of direct SNN training, e.g., quantization errors arising from SNNs' discrete data representations.

### 4.5. Comparisons between binary and ternary SNNs

As discussed in Section 3.3, spiking networks can utilize either binary spikes (Diehl et al., 2015; Hazan et al., 2018) or ternary spikes (Perez-Carrasco et al., 2013; Rathi et al., 2020). In our segmentation networks, we adopt Rathi's approach and use ternary spikes. In this section, we carry out two experiments to investigate the performance differences between binary and ternary SNNs.

The first experiment is an image classification test using the CIFAR-10 dataset. CIFAR-10 consists of 50,000 training images and 10,000 test images, each with a resolution of 32 × 32 pixels and three-color channels (RGB). The dataset is composed of ten distinct classes, including categories such as airplanes, cars, birds, cats, and dogs. We adopt VGG-5 network as the classification network. We trained the network with two setups: the input images are normalized into the ranges [0, 1] and [−1, 1], which we call *without zero-mean* and *with zero-mean* transformations, respectively. After converting to SNNs, the first network becomes a binary SNN, and the second is a ternary spiking network. The classification accuracies are summarized in Table 4. The results demonstrate that SNNs using 3-value spikes generally perform better than binary SNNs. However, we should note binary SNNs are more straightforward to implement in hardware.

**Table 4:** Comparison of binary SNNs and ternary SNNs on an image classification task.

**Normalization**	**ANN**	**SNN inputs**	**Converted SNN accu**.	**Fine-tuned SNN accu**.
With zero-mean	**0.874**	Ternary {−1, 0, 1}	**0.839**	**0.8624**
Without zero-mean	0.867	Binary {0,1}	0.6852	0.8272

*Accu*. is an abbreviation of accuracy.

We conduct the second experiment on semantic segmentation using the same hippocampus data. Similar to the classification experiment, the input data can be normalized into [0, 1] or [−1, −1], which corresponding to binary SNNs and ternary SNNs after conversion. The segmentation accuracies of different models are summarized in Table 5. The results demonstrate the same trend that ternary SNNs generally outperform binary SNNs. This may imply that the performance of ternary networks may degrade slightly if switching to binary networks and deployed onto real hardware.

**Table 5:** Comparison of binary SNNs and ternary SNNs on hippocampus segmentation.

**Normalization**	**ANN accuracy**		**Converted SNN accuracy**		**Fine-tuned SNN accuracy**	
	**2D**	**3D**	**2D**	**3D**	**2D**	**3D**
With zero-mean	**0.8111**	**0.8585**	**0.6482**	**0.7311**	**0.8148**	**0.8526**
Without zero-mean	0.8069	0.8260	0.3107	0.2566	0.8025	0.8403

The models are trained with dice loss on one-fold of the data.

## 5. Discussions and conclusions

SNNs are increasingly being applied in computer vision (CV) tasks, and their performance has steadily improved with the developments of new conversion and optimization solutions. ANN-SNN conversion techniques empower researchers to directly leverage the well-developed ANN training techniques, as well as the pretrained networks. However, it poses a performance ceiling for the converted SNNs. Direct training techniques, on the other hand, address the non-differentiability issue through surrogate gradients and train SNN from scratch. However, training SNNs is more difficult than that of ANNs, often requiring significant time and resources.

In our design, we employ a three-stage training method that combines the benefits from both conversion and direct training. Our pipeline leverages the conversion technique to obtain a good initial state of the SNN from the ANN, without exhaustive full-conversion attempts. Then, our model utilizes SNN training to significantly enhance the performance of the incompletely converted SNN, reaching a level comparable to the original ANN.

In our three-stage pipeline, SNNs are initialized from ANNs with incomplete updates, prior to the fine-tuning step. A significant accuracy drop can be observed from the converted SNNs. While this degradation should be considered as an inevitable result of incomplete conversion, it is worth investigating whether there are other ANN-SNN mapping techniques that could deliver equivalent efficiency but with much improved accuracy.

In this work, the target task and networks focus on segmetic segmentation. We take the human hippocampus as the target structure and demonstrate the effectiveness of our approach through experiments on ADNI data. While U-net was originally developed as a solution for image segmentation, theoretically, it is applicable for any task that seeks to seek mappings between paired signal sources. Hence, it's well-suited and extensively used in image modality conversion, super-resolution, speech signal denoising and enhancement, and generative AI, including image generation models. In this regard, our SNN U-net fine-tuning pipeline has the potential to make a significant impact across a wide range of applications.

This study focuses on algorithmic innovations that aim to enhance segmentation accuracy while ensuring efficiency in a neuromorphic system setting. The networks are developed and tested all within simulation settings. Our current effort includes deploying our pipeline onto real neuromorphic hardware, such as Intel's Loihi 2. This would facilitate real-time system evaluations and the performance of energy and latency analyses.

## Data availability statement

The original contributions presented in the study are included in the article/supplementary material, further inquiries can be directed to the corresponding author.

## Author contributions

YY: Conceptualization, Data curation, Formal analysis, Investigation, Methodology, Software, Visualization, Writing—original draft, Writing—review & editing. MB: Conceptualization, Data curation, Software, Writing—original draft. NA: Conceptualization, Methodology, Software, Writing—original draft. TS: Data curation, Software, Writing—original draft, Writing—review & editing. AK: Conceptualization, Methodology, Writing—review & editing. CS: Conceptualization, Data curation, Funding acquisition, Methodology, Project administration, Writing—original draft. TB: Conceptualization, Writing—original draft. JL: Conceptualization, Data curation, Formal analysis, Funding acquisition, Investigation, Methodology, Project administration, Resources, Software, Supervision, Validation, Visualization, Writing—original draft, Writing—review & editing.
